# Supplementary material for: PLGA-Encapsulated Haemonchus contortus Antigen ES-15 Augments Immune Responses in a Murine Model
Source: Vaccines (Basel). 2023 Nov 30;11(12):1794. doi: 10.3390/vaccines11121794 (PMC10748113; doi:10.3390/vaccines11121794)
Supplement: Supplementary file 1 [file vaccines-11-01794-s001.zip › vaccines-2694075-supplementary.pdf]

Supplementary Figure S1:

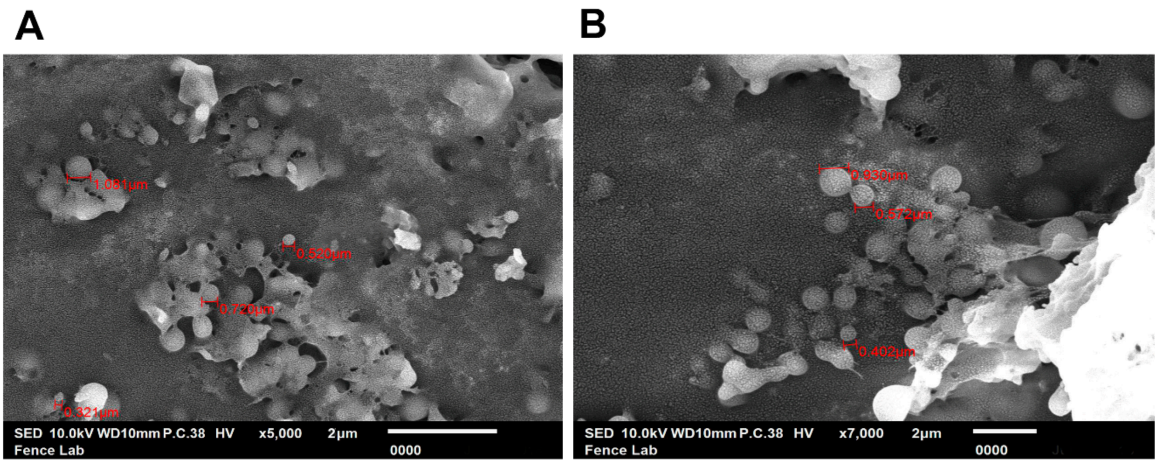

Figure S1 Scanning electron microscopic images of PLGA NPs at different concentrations of PVA. The scale bar is 2 µm.
Notes: (**A**) 1 % PVA. (**B**) 4 % PVA.

Abbreviations: NPs, nanoparticles; PVA, polyvinyl alcohol.

Supplementary Table S1. Nature and composition of the different materials injected into ICR mice to evaluate the type of immune response.

Groups	Inoculations	Injection at Day 0	Purpose
1	PBS	1	Blank control
2	pET-32a	1	Negative control
3	PLGA NPs	1	To compare PLGA NPs
4	rHcES-15	1	To determine immunogenicity of rHcES-15
5	rHcES-15+PLGA	1	To determine immunogenicity of rHcES-15 with the adjuvant activity of PLGA NPs
